# Supplementary material for: A Systematic Review and Meta-Analysis of Seasonal Influenza Vaccination of Health Workers
Source: Vaccines (Basel). 2021 Sep 29;9(10):1104. doi: 10.3390/vaccines9101104 (PMC8537688; doi:10.3390/vaccines9101104)
Supplement: Supplementary file 1 [file vaccines-09-01104-s001.zip › vaccines-1316662-supplementary.pdf]

Supplementary materials

Figure S1. Overall quality assessment of randomized controlled trials included in this study.

Figure S2a. Subgroup analysis of incidence of lab-confirmed influenza regarding conducted country.

Figure S2b. Subgroup analysis of incidence of lab-confirmed influenza regarding study population.

Figure S2c. Subgroup analysis of incidence of lab-confirmed influenza regarding study design.

Figure S2d. Subgroup analysis of incidence of lab-confirmed influenza regarding published year.

Figure S2e. Subgroup analysis of incidence of lab-confirmed influenza regarding average age of experimental groups.

Figure S3a. Subgroup analysis of ILI regarding study design.

Figure S3b. Subgroup analysis of ILI regarding follow-up period.

Figure S4. Sensitivity analysis regarding the outcome of the incidence of lab-confirmed influenza.

Figure S5. Sensitivity analysis regarding the outcome of the incidence of ILI.

Figure S6. Sensitivity analysis regarding the outcome of absenteeism rate.

Figure S7. Sensitivity analysis regarding the outcome of workdays lost per person.

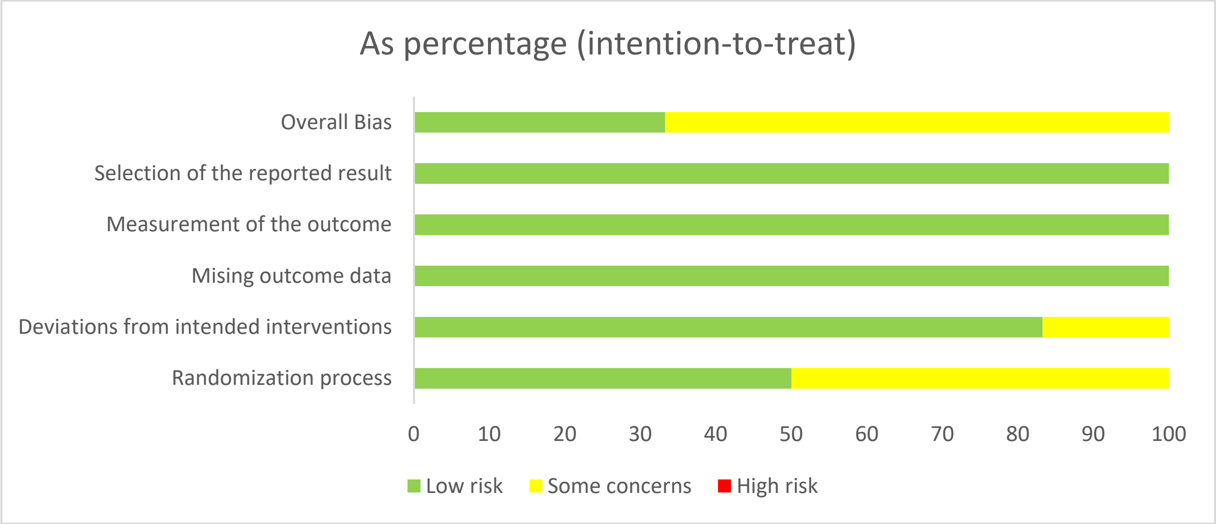

Figure S1. Overall quality assessment of randomized controlled trials included in this study.

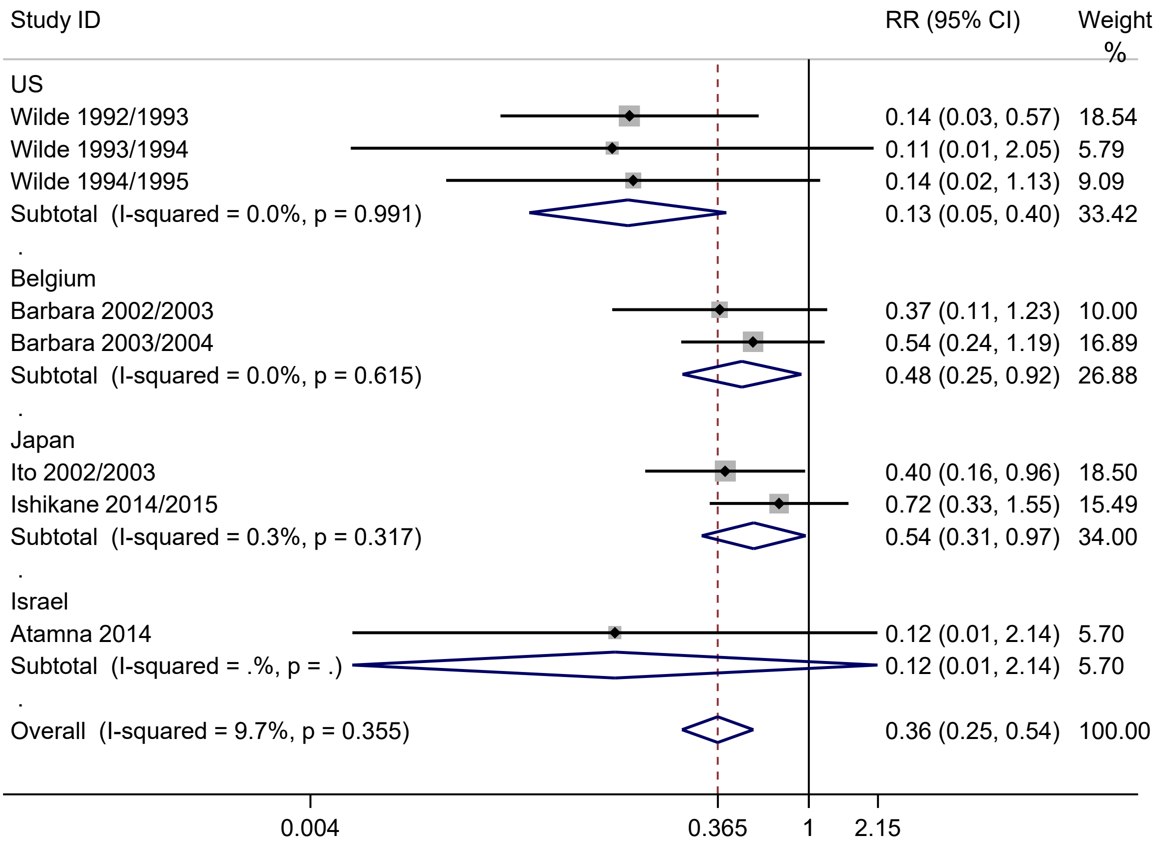

Figure S2a. Subgroup analysis of incidence of lab-confirmed influenza regarding conducted country. ID, identification; RR, risk ratio; RCT, randomized controlled trial.

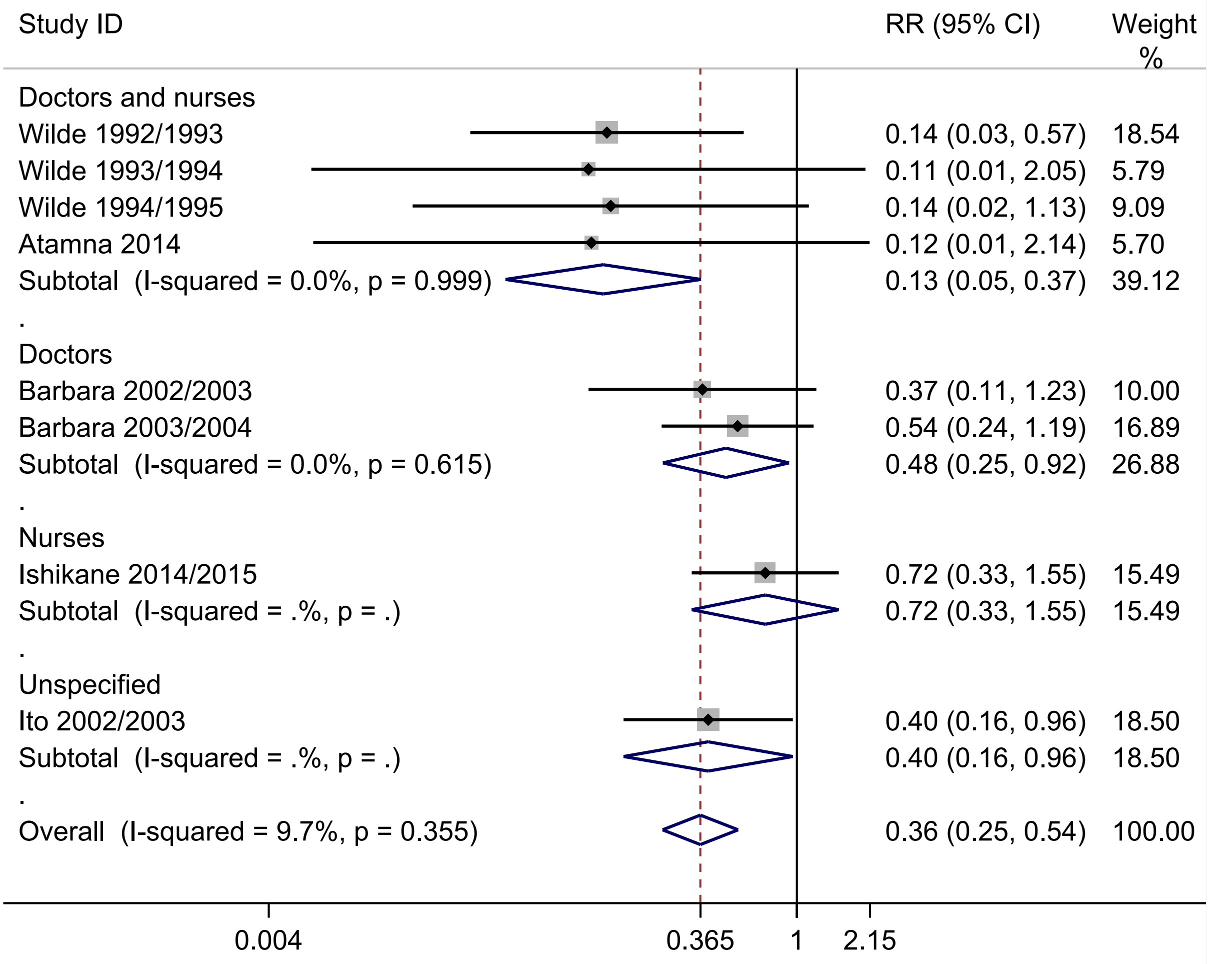

Figure S2b. Subgroup analysis of incidence of lab-confirmed influenza regarding study population. ID, identification; RR, risk ratio; RCT, randomized controlled trial.

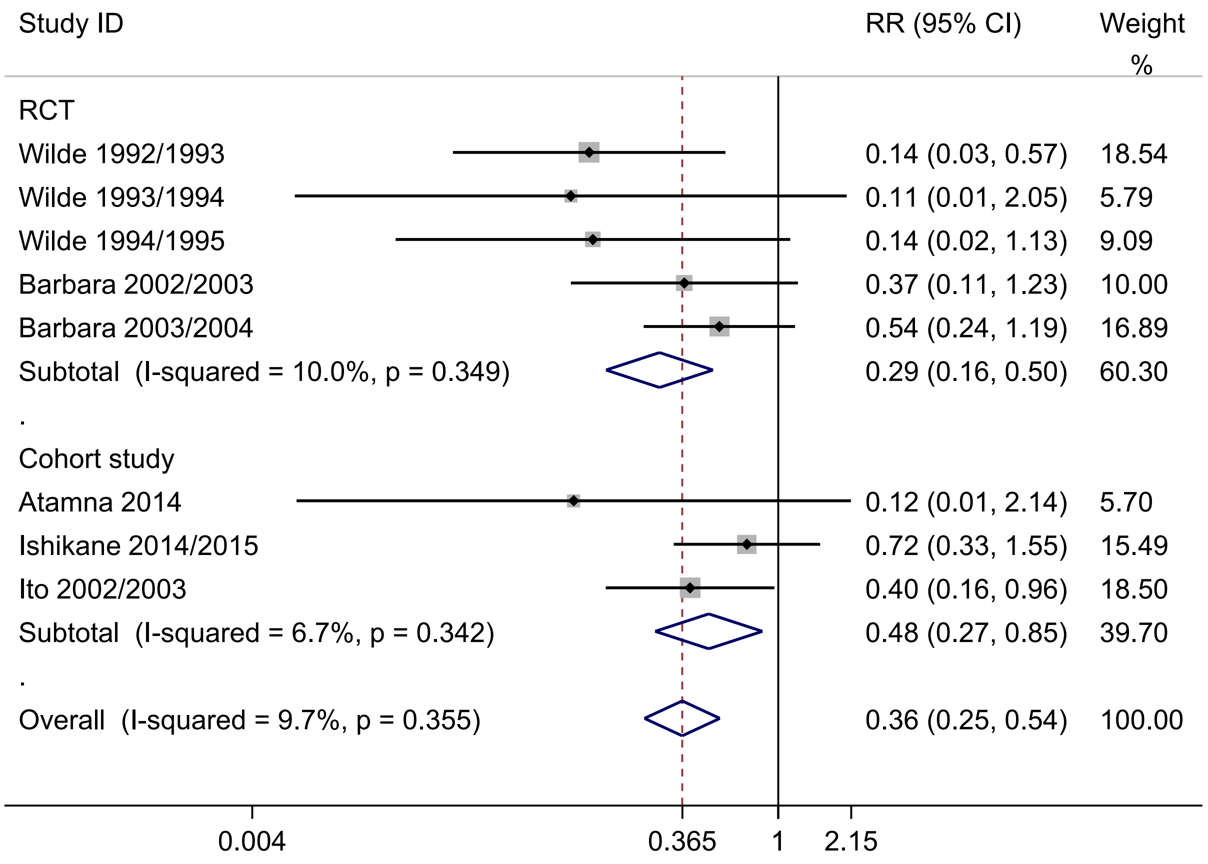

Figure S2c. Subgroup analysis of incidence of lab-confirmed influenza regarding study design. ID, identification; RR, risk ratio; RCT, randomized controlled trial.

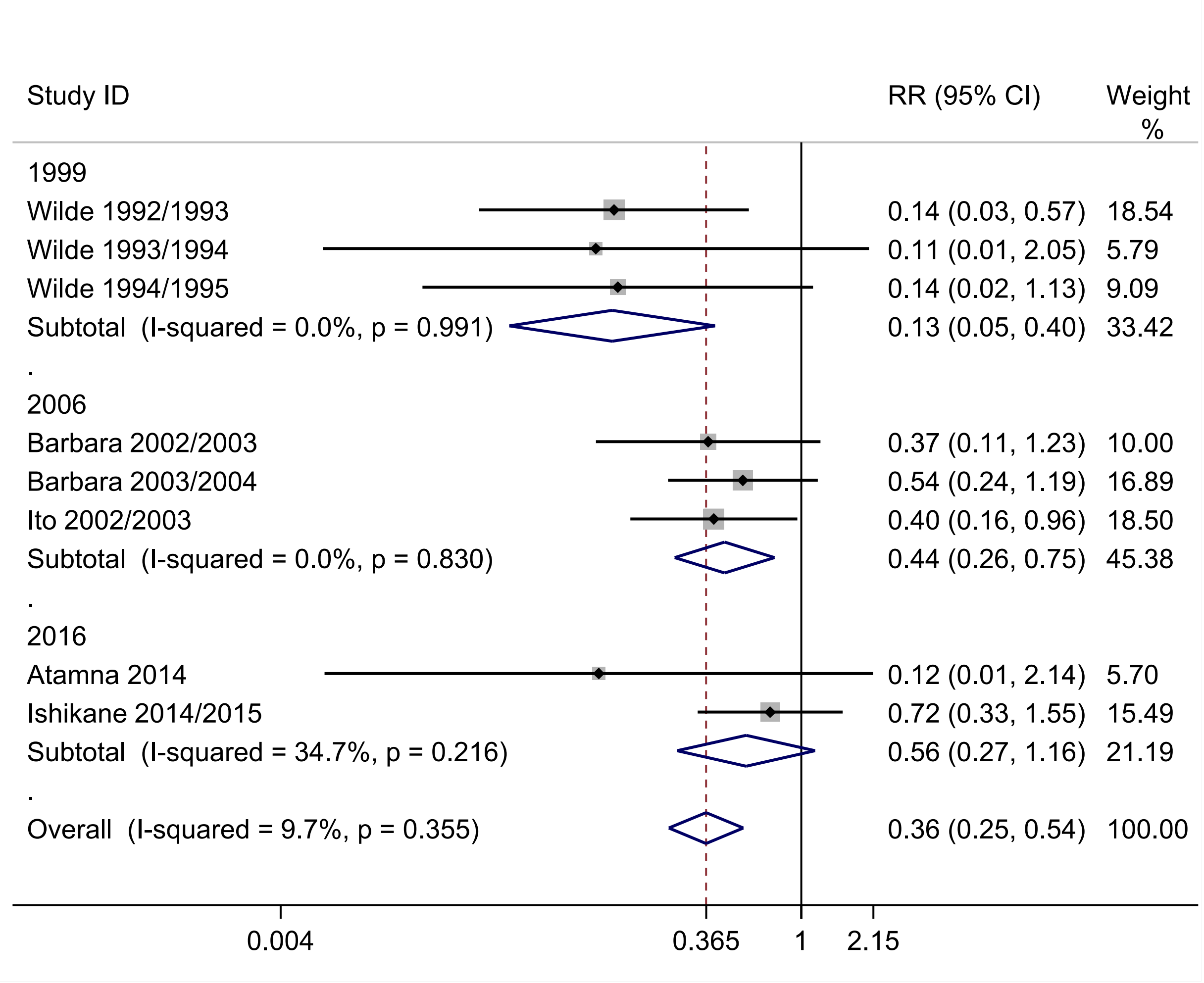

Figure S2d. Subgroup analysis of incidence of lab-confirmed influenza regarding published year. ID, identification; RR, risk ratio; RCT, randomized controlled trial.

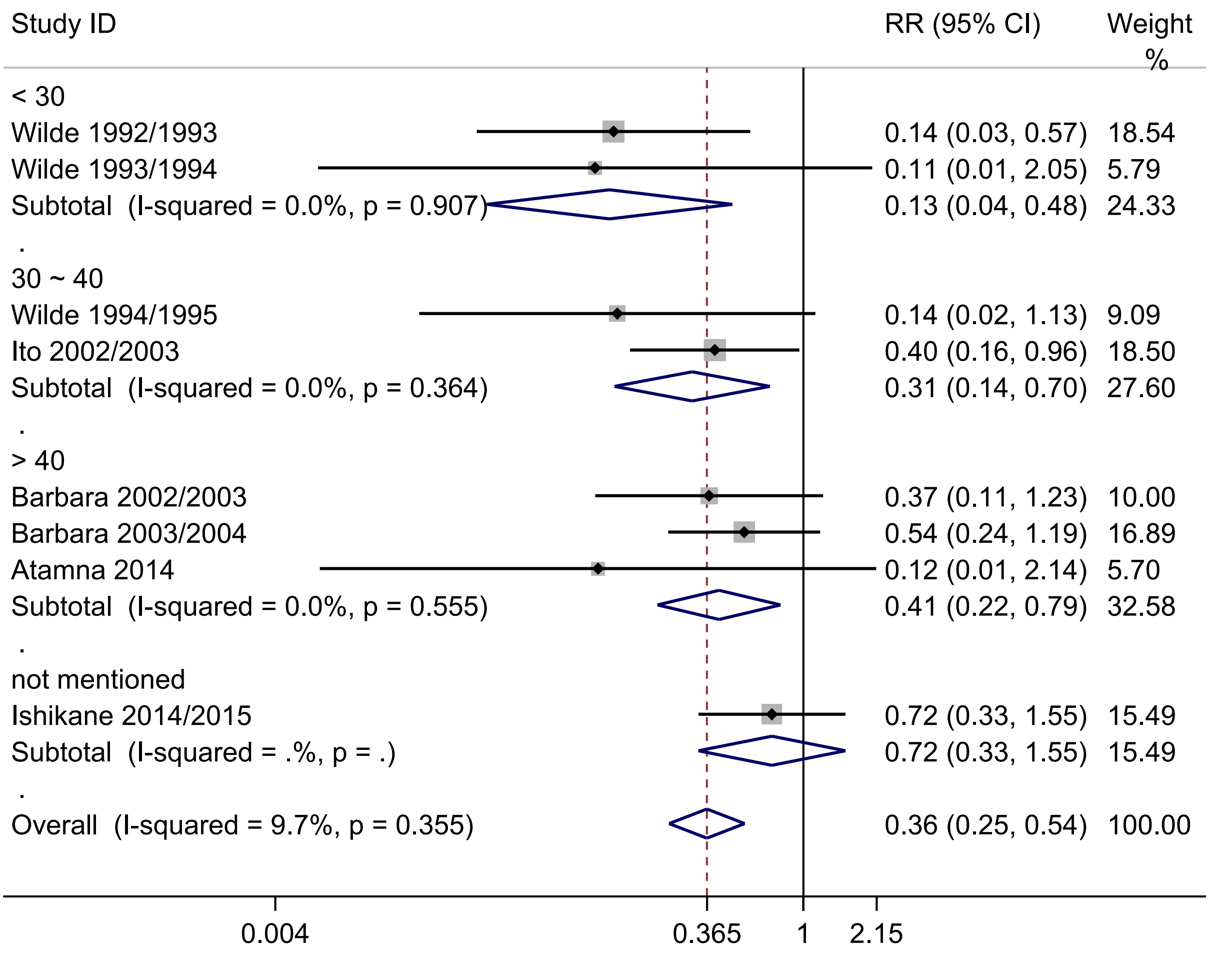

Figure S2e. Subgroup analysis of incidence of lab-confirmed influenza regarding average age of experimental groups. ID, identification; RR, risk ratio; RCT, randomized controlled trial.

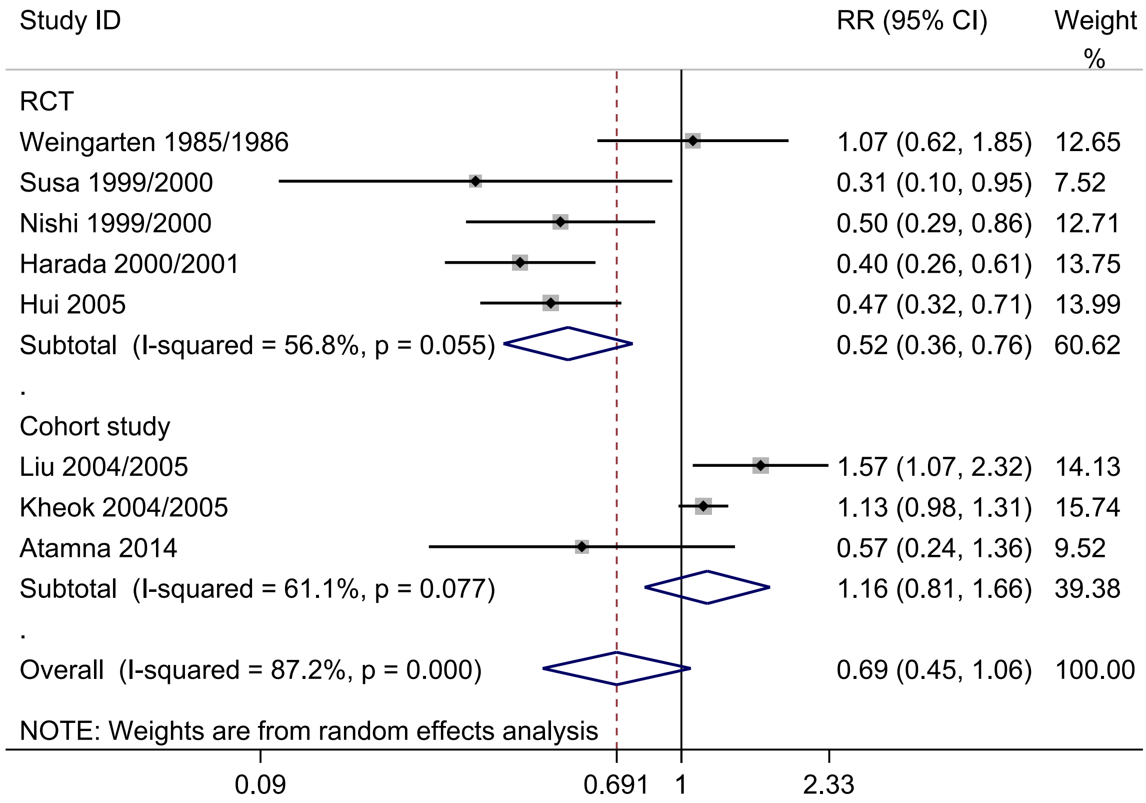

Figure S3a. Subgroup analysis of ILI regarding study design. ILI, influenza-like illness; ID, identification; RR, risk ratio; RCT, randomized controlled trial.

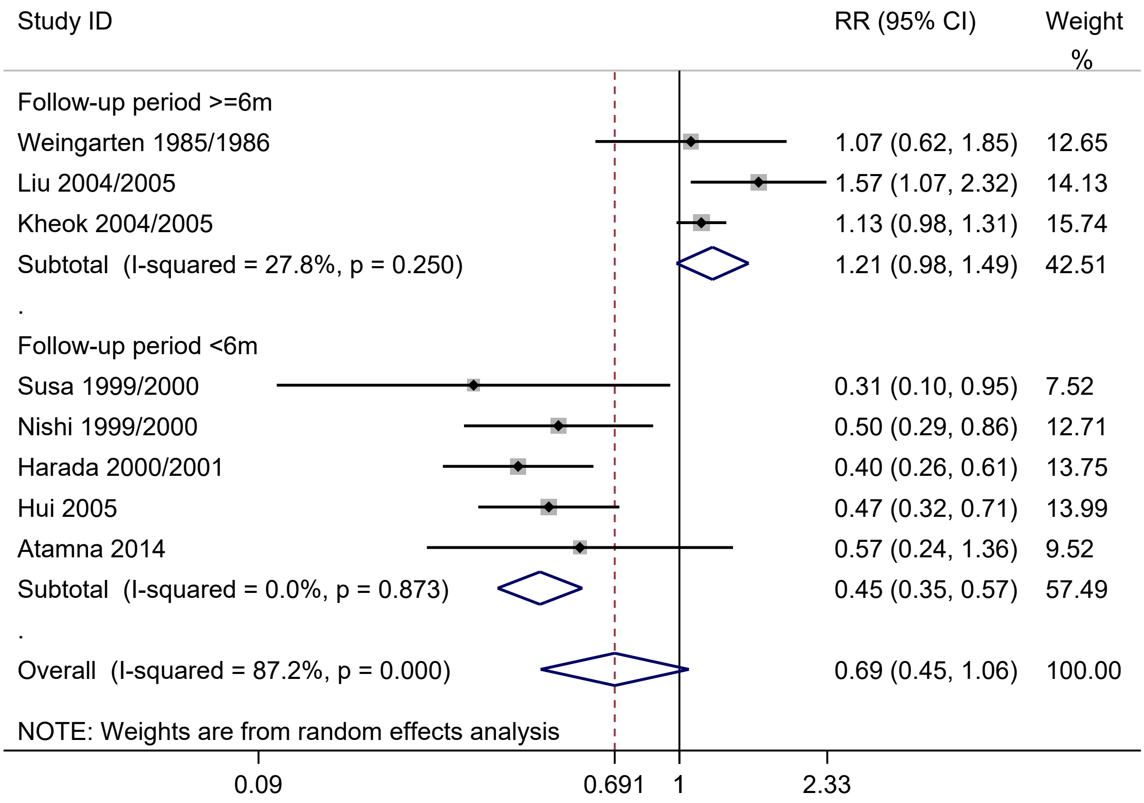

Figure S3b. Subgroup analysis of ILI regarding follow-up period. ILI, influenza-like illness; ID, identification; RR, risk ratio; RCT, randomized controlled trial.

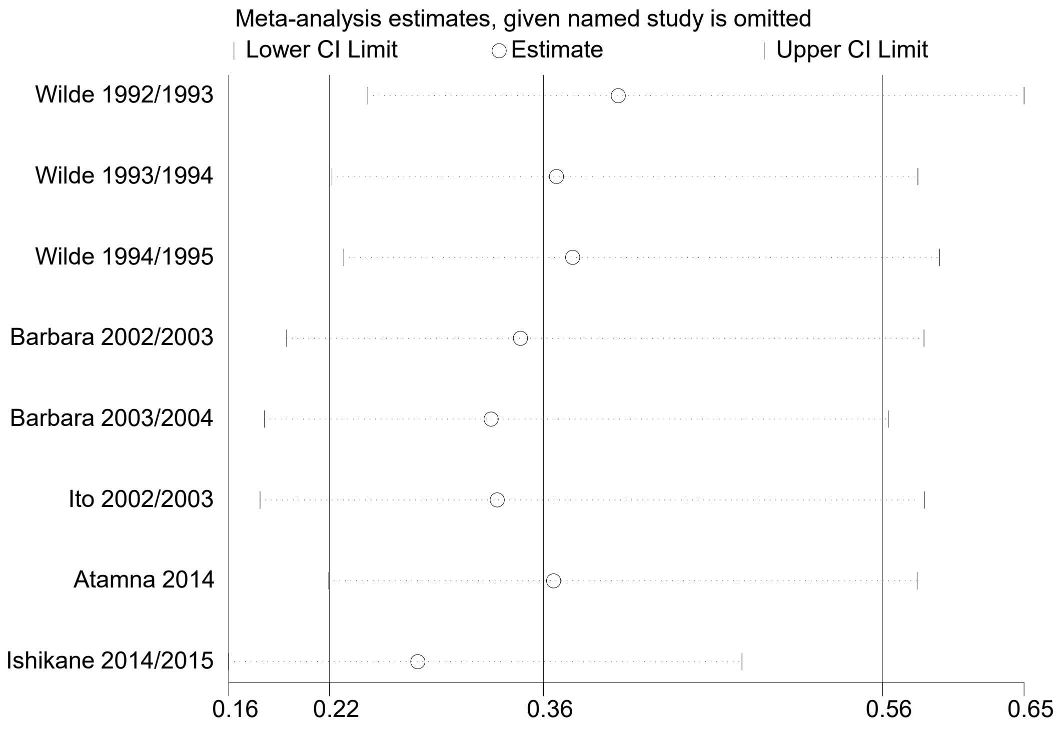

Figure S4. Sensitivity analysis regarding the outcome of the incidence of lab-confirmed influenza.

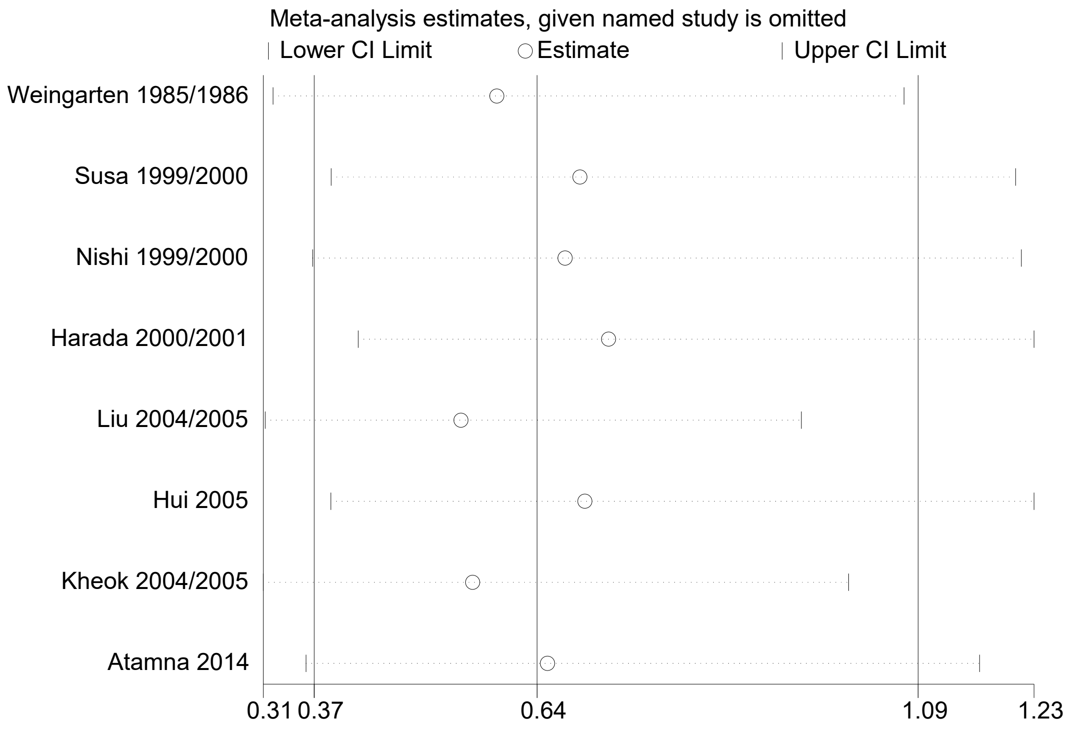

Figure S5. Sensitivity analysis regarding the outcome of the incidence of ILI. ILI, influenza-like illness.

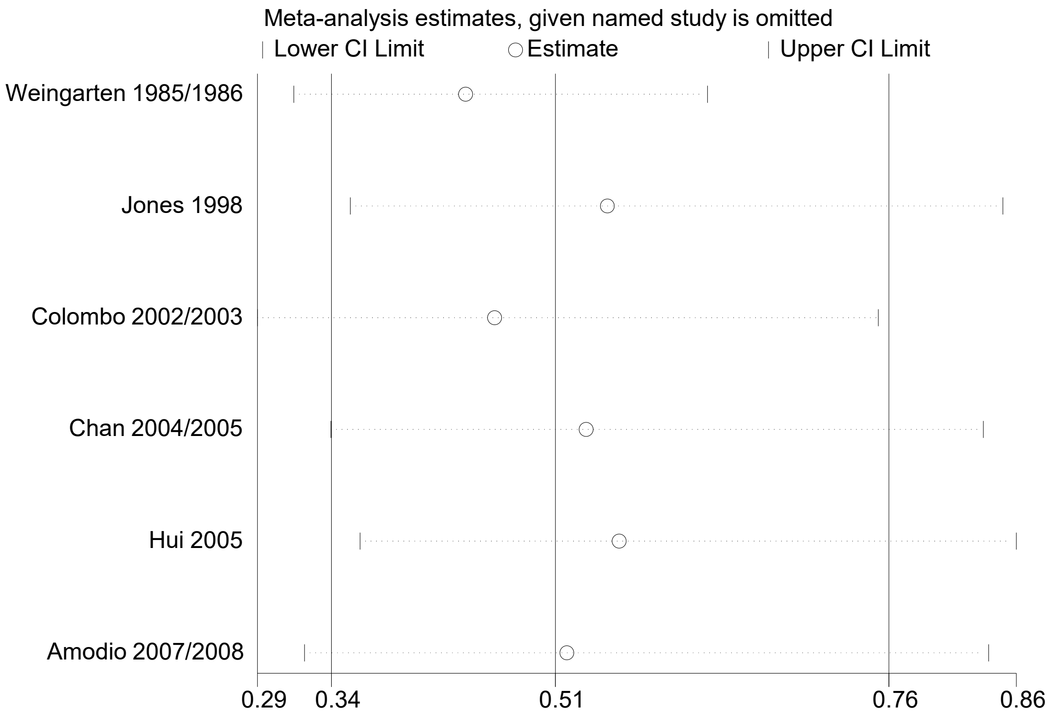

Figure S6. Sensitivity analysis regarding the outcome of absenteeism rate.

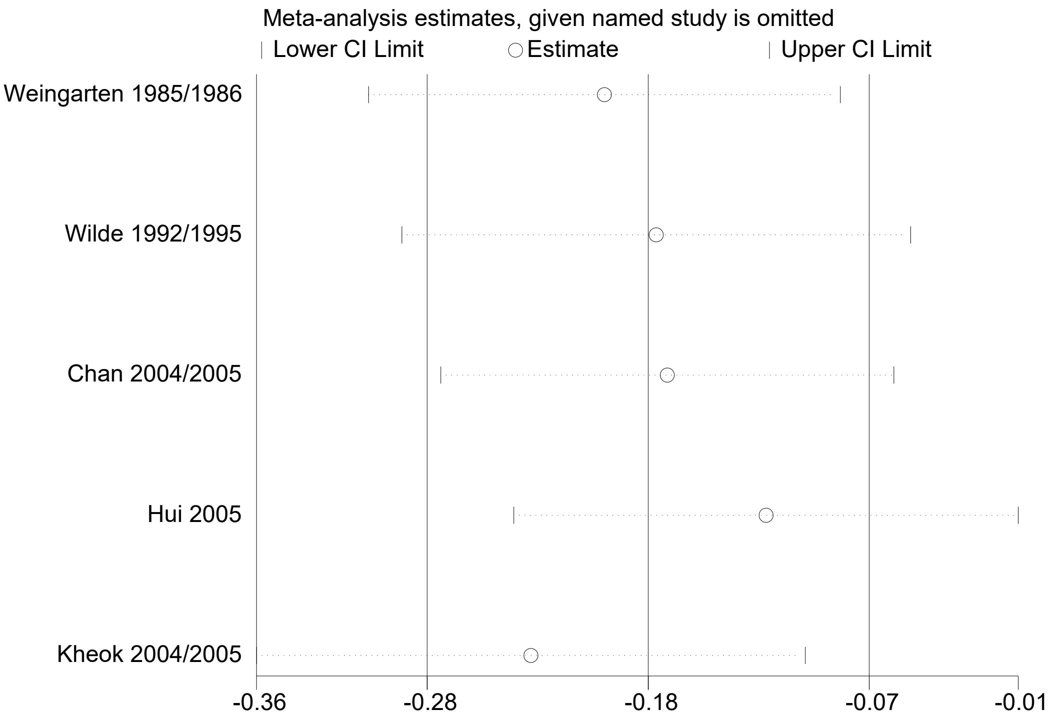

Figure S7. Sensitivity analysis regarding the outcome of workdays lost per person.
